# Optimized Replication of ADC-Based Particle Counting Algorithm with Reconfigurable Multi-Variables in Pseudo-Supervised Digital Twining of Reference Dust Sensor Systems

**DOI:** 10.3390/s23125557

**Published:** 2023-06-14

**Authors:** Seungmin Lee, Jisu Kwon, Daejin Park

**Affiliations:** School of Electronic and Electrical Engineering, Kyungpook National University, Daegu 41566, Republic of Korea; lsm1106@knu.ac.kr (S.L.); kjisu96@knu.ac.kr (J.K.)

**Keywords:** digital twin, dust sensing, particle count, ADC filter, embedded device

## Abstract

As the application fields for digital twins have expanded, various studies have been conducted with the objective of optimizing the costs. Among these studies, research on low-power and low-performance embedded devices has been implemented at a low cost by replicating the performance of existing devices. In this study, we attempt to obtain similar particle count results in a single-sensing device replicated from the particle count results in a multi-sensing device without knowledge of the particle count acquisition algorithm of the multi-sensing device. Through filtering, we suppressed the noise and baseline movements of the raw data of the device. In addition, in the process of determining the multi-threshold for obtaining the particle counts, the existing complex particle count determination algorithm was simplified to make it possible to utilize the look-up table. The proposed simplified particle count calculation algorithm reduced the optimal multi-threshold search time by 87% on average and the root mean square error by 58.5% compared to existing method. In addition, it was confirmed that the distribution of particle count from optimal multi-thresholds has a similar shape to that from multi-sensing devices.

## 1. Introduction

A digital twin replicates real-world environments and simulates prediction results using a computer. The applications of digital twins are expanding throughout the industry owing to their advantages such as safety, repeatability, and the low cost of predicting results [1]. Digital twins are widely used in manufacturing to predict the results of a product, and recently, the scope of autonomous driving has expanded through the digitization of cities [2,3,4,5,6,7,8].

In particular, Industry 4.0 further emphasizes the importance of digital twins in smart factories that have intelligent production systems [9,10,11,12]. Additionally, with the increase in the average life expectancy of people, interest in health is increasing. Recently, due to respiratory diseases caused by the coronavirus, interest in air quality among living environments is on rise [13,14,15].

Dust-sensing devices digitize and provide dust concentration information in the air. The use of a digital twin of dust concentration is important in minimizing the occurrence of defects in ultra-fine processes in the industrial field [16], and in the case of buildings, it is used for periodic internal air circulation [17,18]. The importance of digital twins in the continuous management and conservation of energy consumption, particularly in relation to the heating, ventilation, and air conditioning systems of buildings, is consistently increasing [19]. In daily life, the increase in indoor fine dust concentration due to air pollution is measured and used to establish an IoT environment linked to the automatic operation of air purifiers and fans.

Digital-twinned dust sensing systems are used in both industry and daily life, and their field of application is expanding [20,21]. Accordingly, to cover long-term operation and wide measurement areas, existing devices are being replaced by low-power and low-cost embedded devices, and existing algorithms are being improved and optimized to be made suitable for low-memory embedded devices [22,23].

In this study, we minimized the cost by replicating the particle count (PC) of an existing dust sensing device. The existing device uses multiple sensors to measure the number of particles according to the size of dust. In this study, we attempt to replicate the performance of a multi-sensor device (reference device) through a single-sensor device (test device) to minimize the power consumption and reduce the cost of the dust sensing system.

To this end, this study was conducted in three parts: (1) analog-to-digital converter (ADC) filter design, (2) multi-threshold search, and (3) PC similarity analysis.

First, the light scattered by the dust was measured in terms of voltage using a photodiode. The larger the dust particle, the more light is scattered, resulting in a higher voltage. The change in the magnitude of the voltage measured by the photodiode is small. Thus, if the baseline of the ADC fluctuates owing to the power supply noise, an error occurs in the PC calculation. To improve this, in this study, we minimized the effect of power noise by detecting baseline fluctuations using an average filter and then removing them. In addition, because the test device is vulnerable to noise, an average filter was additionally applied to suppress noise.

After filtering the ADC, determining a multi-threshold for measuring the PC according to the size of the dust particle for the filtered ADC is necessary. In general, it is to generate a look-up table (LUT) for a PC measurement algorithm because the threshold used in the PC measurement process is not independent of other thresholds. Therefore, the searching time for optimal multi-thresholds increases exponentially with the number of dust size categories. Furthermore, the general PC measuring algorithm considers the width of the voltage pulse in the ADC. This makes the algorithm more complex and makes it difficult to generate LUTs, which further increases the searching time for optimal multi-thresholds. The simplification of optimal multi-threshold searching algorithm is required because run-time execution is important in digital twinning using light-weight embedded devices. In this study, we improved the searching time and root mean square error (RMSE) using LUTs by simplifying various variables used for the PC measurement. We reduced search time by 87% and RMSE by 58.5% compared to the existing method.

In searching for the optimal multi-threshold, the suitability of the given multi-threshold is determined by comparing the PCs from the test device (TPCs) with that from the reference device (RPCs). However, the TPCs are generally lower than the RPCs because the test device is a single-sensor device. Thus, comparing the two sets of PC data was difficult. To solve this, we normalized RPCs and TPCs in two steps. Firstly, we normalized RPCs and TPCs according to the maxima of RPC0.3 and TPC0.3, which are generally the highest values between PCs. This process normalizes the scale between the RPCs and TPCs. After the first normalization, a secondary normalization is performed for each RPC and TPC with the corresponding maxima of the RPCs. This process normalizes the internal scales of RPCs and TPCs. After normalization, the similarity was compared using the RMSE.

By repeating the update of the multi-threshold and similarity measurement, the optimal multi-threshold with the highest similarity are determined. Then, we can obtain the TPCs using the optimal multi-threshold that replicates the RPCs.

**Figure 1:**
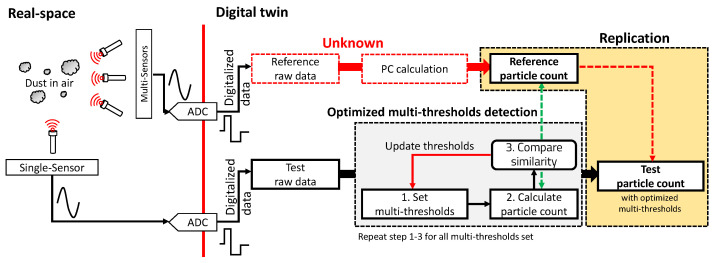
Digital twin in dust sensing.

Figure 1 is a schematic of the proposed method.

In this manner, without a known ADC and PC measuring algorithm for the reference device, the optimal multi-thresholds are determined such that the TPCs replicate the RPCs.

The remainder of this paper is organized as follows. Section 2 introduces the structure of the dust-sensing system and explains the ADC filtering. Section 3 explains the TPCs acquisition processes using optimal multi-thresholds. After confirming that the RPCs are replicated well through experiments on the acquisition of TPCs in Section 4, concluding statements are provided in Section 5.

## 2. Dust Sensing System

### 2.1. Light Scattering Method

PM encompasses particles of sulfate, black carbon, dust from erosion, pollen, and so on [24]. Various methods are available for measuring dust concentration. Existing representative dust measurement methods for PM2.5 include the gravimetric method and the beta-ray absorption method [25]. The gravimetric method manually measures the weight of the collected dust, while the beta-ray absorption method automatically measures the concentration using the amount of beta-rays absorbed by the dust.

Although the gravimetric method is accurate, it requires considerable time to collect dust and has the disadvantage of needing to maintain a consistent temperature and humidity during the measurement process. The beta-ray absorption method measures the dust concentration at intervals of one hour, but its accuracy is relatively lower compared to the gravimetric method. This measurement method is suitable for confirming the concentration of dust in everyday life on a daily or hourly basis; however, it is not suitable for real-time measurements.

Recently, various sensor-based measurement devices have been developed to overcome the limitations of traditional dust measurement devices, even though their reliability has not been fully achieved. Among these devices, the light scattering method-based measurement device is lightweight, compact, and capable of providing measurements within a short time frame, ranging from one second to one minute [26,27].

Table 1 presents the comparison of characteristics of different dust sensing methods.

The light scattering method has a limitation in that it cannot be used for administrative statistics due to its low accuracy compared to other methods. However, it is the most suitable for digital twinning of dust sensing due to its advantages such as its portability, low power consumption, low cost, and real-time processing.

**Figure 2:**
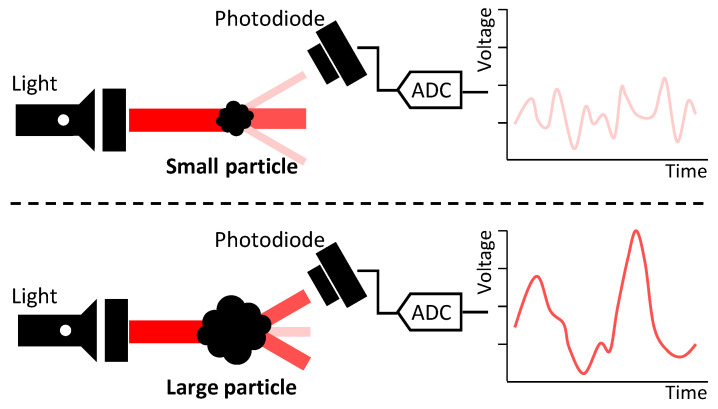
Light scattering method.

The light-scattering method measures the amount of light scattered by dust. Figure 2 illustrates the concept of the light scattering method.

The larger the dust, the more light is scattered, resulting in a higher intensity of the detected light and wider voltage pulses. Based on this characteristic, the PC categories were classified according to the magnitude of the voltage, and the number of samples falling within each category range was counted. The sampling frequencies of the ADC and TPCs used in this study were 50 kHz and 1/6 Hz, respectively. One TPC sample for each PC category was calculated by counting 300,000 ADC samples.

Typically, the number of dust particles based on size is primarily counted for each category of differential PC (dPC), and the actual PC value is subsequently measured by accumulating the dPC values. For instance, dPC0.3 consists of particle counts smaller than 0.5 μm, and the threshold of dPC is determined by the next category of dPC.

Moreover, dPC0.5, dPC1.0, ⋯, and dPC10.0 are calculated in a similar way. PC0.3 is the accumulation of dPC0.3 to dPC10.0, and PC0.5 is the accumulation of dPC0.5 to dPC10.0, as shown in (1):

(1) $$\begin{array}{ccc} PC0.3 & = & dPC0.3+dPC0.5+\cdots +dPC10.0\\ PC0.5 & = & dPC0.5+dPC1.0+\cdots +dPC10.0\\ \vdots \\ PC10.0 & = & dPC10.0 \end{array}$$

Thus, PC0.3 ≥ PC0.5 ≥⋯≥ PC10.0.

### 2.2. ADC Filtering

**Figure 3:**
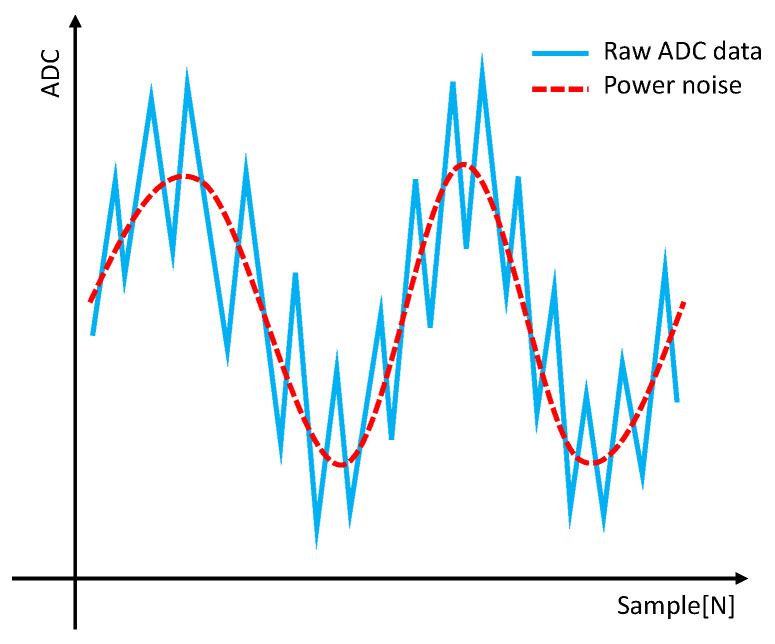
ADC raw data and baseline fluctuation noise.

The light scattered by dust was measured using a photodiode. The voltage measured increased as the size of the dust particles increased. The PC is categorized into various categories based on the size of the dust, such as PC0.3, PC0.5, and PC1.0. To obtain the PC for each category, thresholds are required. In general, a global threshold was used assuming no changes in environmental conditions. However, when the global threshold is used, variations in the baseline caused by power supply noise can lead to erroneous detection. Additionally, it becomes challenging to use the existing global threshold when there are overall voltage fluctuations due to external environmental factors during the measurement. To address these issues, baseline variations and bases were eliminated, creating an environment suitable for the application of global thresholds. Figure 3 illustrates the raw ADC data, showing the fluctuations in the baseline.

As shown in Figure 3, baseline fluctuations caused by power supply noise were observed. These fluctuations posed challenges when detecting particles based on the global threshold. Excessive particle detection occurred when the baseline was low, while all samples exceeded the threshold when the baseline was high, resulting in no particle detection. Therefore, a preprocessing step is necessary to suppress baseline fluctuations.

Baseline fluctuations are commonly mitigated using a high-pass filter. Another approach involves acquiring baseline information through a low-pass filter and then removing the low-pass-filtered signal to suppress baseline fluctuations. In this study, we designed a suitable filter, considering both finite impulse response (FIR) filters and infinite impulse response (IIR) filters, to effectively suppress baseline fluctuations.

#### 2.2.1. FIR Average Filter

The test device used in this study can apply to an eight-tap FIR filter. Based on this, we apply a seven-tap moving average filter as in (2):

(2) $$y\left[n\right]=\sum_{i=0}^{6}\frac{1}{7}x\left[n-i\right]$$

**Figure 4:**
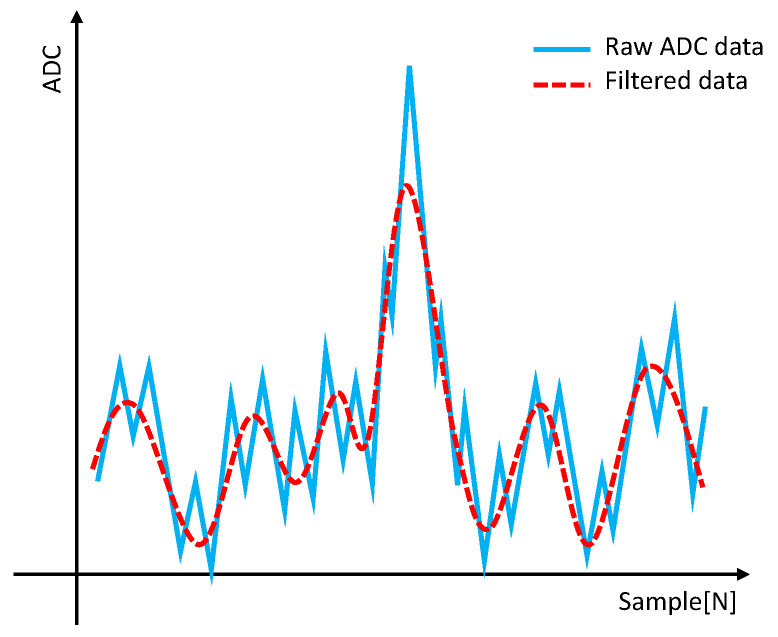
Input and filtered ADCs using 7-tap FIR moving average filter.

Figure 4 illustrates the input and filtered ADCs.

As a result, the FIR moving average filter, which uses a small number of taps, is highly responsive to baseline changes and can be influenced by high ADC samples. Consequently, it has a disadvantage in preserving high ADC samples generated by a large amount of dust.

On the other hand, if the window size of the moving average is increased, the filter becomes less sensitive to baseline changes, leading to improved preservation of high ADC samples. However, increasing the number of taps in the FIR filter significantly increases the cost, presenting a challenge.

#### 2.2.2. IIR Average Filter

The IIR filter offers the advantage of achieving a similar effect to that of a large number of taps in an FIR filter, even with a small number of taps. The transfer function of the IIR filter is defined using the Z-transform, as shown in (3):

(3) $$H\left(z\right)=\frac{Y(z)}{X(z)}=\frac{\sum_{i=0}^{P}b_{i}z^{-i}}{\sum_{j=0}^{Q}a_{j}z^{-j}},$$

where *P* is the feedforward filter order, *Q* is the feedback filter order, $b_{i}$ represents the feedforward filter coefficients, and $a_{j}$ represents the feedback filter coefficients.

In this paper, we designed a second-order IIR filter to produce a similar result to that of an FIR filter with a window size of 50, as shown in (4):

(4) $$\begin{array}{ccc} b_{i} & = & \left[0.02,0.000417,0.000417\right]\\ a_{j} & = & \left[1,-0.97917,0\right] \end{array}$$

**Figure 5:**
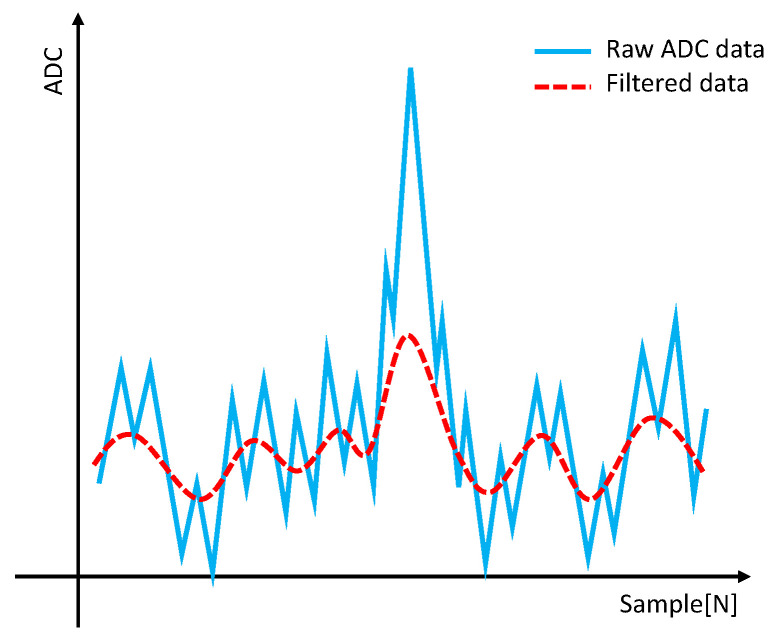
Input and filtered ADCs using second-order IIR filter.

Figure 5 illustrates the experimental results using the IIR filter.

The results of the 49-tap FIR filter using MATLAB were compared, and it was confirmed that the results of the second-order IIR filter were sufficiently similar.

#### 2.2.3. Composite FIR and IIR Filters

From the previous experiment, it was confirmed that the IIR filter can effectively detect baseline fluctuations, even with a small number of taps. In order to further improve the filtering performance, a 4-tap lowpass filter was applied to the FIR filter to suppress extreme high-frequency noise signals. This means that the FIR filter suppresses the high-frequency component, while the IIR filter captures the baseline and suppresses it, resulting in an overall effect similar to a bandpass filter that suppresses the low-frequency component.

### 2.3. General PC Calculation Algorithm

From the filtered ADC data, the number of voltage pulses was counted using each threshold to measure each dPC. Subsequently, each PC was obtained by accumulating the corresponding dPC values. Therefore, multiple thresholds are required, one for each category of PC.

In general, the amount of light scattering increases with the size of the dust, resulting in higher voltage measurements on the photodiode. This indicates a larger amount of dust being observed. As a result, the ADC threshold gradually increases depending on the size of the dust: Thr0.3 ≤ Thr0.5 ≤⋯≤ Thr10.0. Therefore, when searching for a threshold, the search can be performed within a range larger than the threshold of the previous category. Using a LUT can also be effective in minimizing overlapping operations since previously calculated thresholds often overlap during the multi-threshold search process.

**Figure 6:**
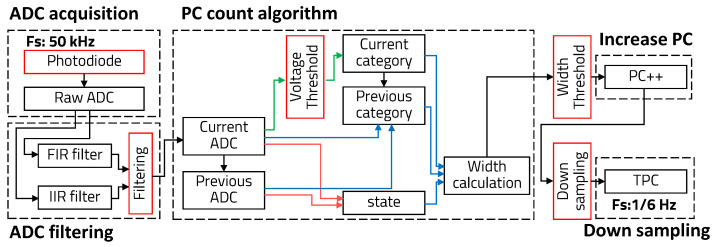
Algorithm scheme of general PC calculation.

However, the general PC calculation algorithm is not simply based on applying the threshold to the ADC values. It takes into account factors such as the change in states (increase or decrease) between previous and current samples, category changes based on the threshold, and the width of the voltage pulse. Figure 6 illustrates the conceptual diagram of the general PC calculation algorithm.

It is necessary to consider not only the optimization of multiple thresholds for the ADC voltage but also multiple thresholds for the pulse width. The measurement results of PC according to these thresholds are not independent of each other. Therefore, applying an LUT to the general PC calculation algorithm becomes difficult.

## 3. Proposed Algorithm

### 3.1. Simplified PC Calculation Algorithm

In this study, we simplified the algorithm by incrementing each PC when the voltage of a sample exceeded a given threshold. In this simplified case, the same PC is obtained regardless of the category for which the threshold is used to calculate the PC. As a result, the PC values obtained according to the thresholds can be generated as an LUT. During the optimal multi-threshold searching process, when calculating the similarity of TPCs for a given set of multi-thresholds, there is no need to calculate the TPCs each time based on the given thresholds. Instead, we can refer to the TPCs in the LUT and compare the similarity with the RPCs.

**Figure 7:**
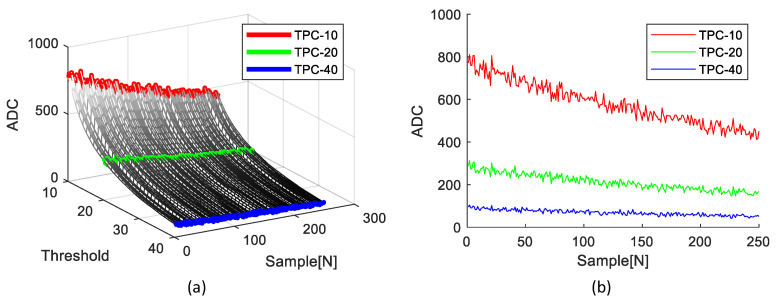
The LUT distribution and the example of TPCs according to thresholds: (**a**) LUT distribution; (**b**) TPCs with thresholds 10, 20, and 40.

Figure 7 illustrates the distributions of the LUT based on different thresholds and an example of the TPC at thresholds 10, 20, and 40.

**Figure 8:**
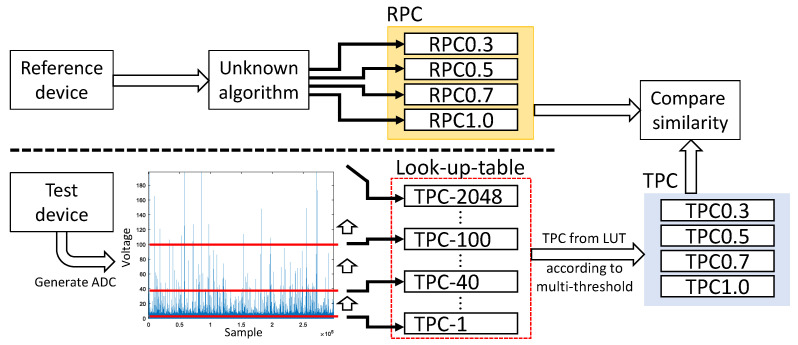
The simplified TPC calculating algorithm using LUTs.

Figure 8 shows the simplified TPCs calculating algorithm using LUT.

Compared to Figure 6, the redundancy of the calculation of the TPCs according to the threshold is removed, resulting in a simplified process.

### 3.2. PC Similarity Measurement

In general, the RMSE is widely used to measure the similarity of two signals.

For two given signals *X* and *Y*, the RMSE is calculated as in (5):

(5) $$RMSE\left(X,Y\right)=\sqrt{\frac{\sum_{i=1}^{N}{\left(x_{i}-y_{i}\right)}^{2}}{N}},$$

where *N* is the length of the two signals.

RMSE is commonly used to measure the similarity between two signals when their scales are similar.

However, comparing the similarity between RPCs and TPCs becomes challenging due to the significant difference in their scales. In such cases, the Pearson similarity method [28] is often employed as an alternative. The Pearson similarity is calculated using (6), which is suitable for comparing signals with different scales:

(6) $$\rho \left(X,Y\right)=\frac{1}{N-1}\sum_{i=1}^{N}\left(\frac{X-\mu_{X}}{\sigma_{X}}\frac{Y-\mu_{Y}}{\sigma_{Y}}\right),$$

where μ and σ represent the mean and standard deviation of the signal, respectively.

The Pearson similarity is a robust measure that takes into account baseline movements and scale changes between signals. It achieves this by normalizing the signals through the subtraction of their mean values and division by their standard deviations. This normalization process helps to mitigate the impact of baseline shifts and variations in scale, allowing for a more accurate comparison of the signals’ similarity.

In this study, instead of comparing two single-channel signals, it is necessary to compare multiple multi-channel signals corresponding to the number of PC categories. As a result, normalizing RPCs and TPCs using the Pearson similarity becomes challenging.

In this study, we calculate the RMSE after normalizing the scales of RPCs and TPCs through two-step normalization using (7) and (8):

(7) $$\begin{array}{ccc} RPC0.3^{\prime } & = & RPC0.3/max(RPC0.3)\\ TPC0.3^{\prime } & = & TPC0.3/max(TPC0.3)\\ RPC0.5^{\prime } & = & RPC0.5/max(RPC0.3)\\ TPC0.5^{\prime } & = & RPC0.5/max(TPC0.3)\\ \vdots \\ RPC10.0^{\prime } & = & RPC10.0/max(RPC0.3)\\ TPC10.0^{\prime } & = & RPC10.0/max(TPC0.3) \end{array}$$

(8) $$\begin{array}{ccc} RPC0.3^{\prime \prime } & = & RPC0.3^{\prime }/max\left(RPC0.3^{\prime }\right)\\ TPC0.3^{\prime \prime } & = & TPC0.3^{\prime }/max\left(RPC0.3^{\prime }\right)\\ RPC0.5^{\prime \prime } & = & RPC0.5^{\prime }/max\left(RPC0.5^{\prime }\right)\\ TPC0.5^{\prime \prime } & = & RPC0.5^{\prime }/max\left(RPC0.5^{\prime }\right)\\ \vdots \\ RPC10.0^{\prime \prime } & = & RPC10.0^{\prime }/max\left(RPC10.0^{\prime }\right)\\ TPC10.0^{\prime \prime } & = & RPC10.0^{\prime }/max\left(RPC10.0^{\prime }\right) \end{array}$$

**Figure 9:**
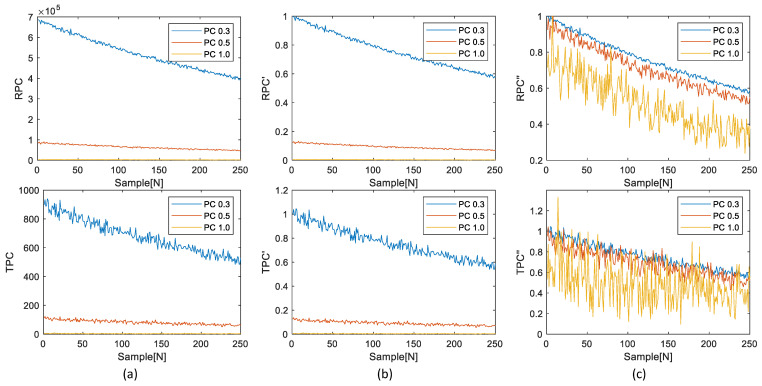
Example of normalization process for calculating RMSE: (**a**) RPCs and TPCs with given multi-thresholds, (**b**) first normalization using maxima of RPC0.3 and TPC0.3, and (**c**) secondary normalization using maxima of RPCs’.

Figure 9 shows the process of primary and secondary normalized RPCs and TPCs for measuring the RMSE.

Equation (7) and Figure 9b depict the first normalization of RPCs and TPCs, denoted as RPCs’ and TPCs’. This first normalization is performed by dividing RPCs and TPCs by their respective maxima, specifically RPC0.3 and TPC0.3. By doing so, the scale difference between the RPCs and TPCs is addressed. Equation (8) and Figure 9c illustrate the secondary normalization of RPCs and TPCs, denoted as RPCs” and TPCs”. This secondary normalization is carried out to address the scale difference within each multi-channel signal, namely RPCs’ and TPCs’. During the secondary normalization process, RPCs’ and TPCs’ are divided by their corresponding maxima in RPCs, allowing the RMSE to be calculated based on the magnitude of RPCs.

After normalizing the RPCs and TPCs, the similarity between them can be accurately measured by utilizing the RMSE metric.

**Figure 10:**
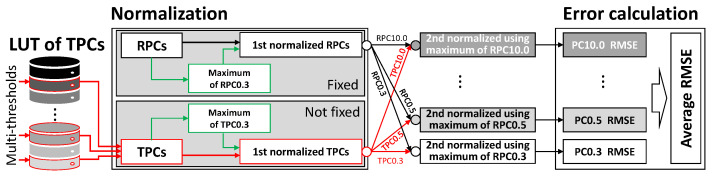
RMSE between RPCs and TPCs using two-step normalization.

Figure 10 shows the process of measuring the RMSE through normalization based on PC0.3.

For the RPCs, fixed data were obtained from the reference device. However, in the case of TPCs, even if the same PCs are obtained from the LUT using the same threshold, the normalized PC values can differ due to variations in the maximum value of PC0.3. As a result, the LUT stores TPCs before normalization and measures their similarity after normalizing the TPCs obtained from the LUT. This approach allows for accurate comparison and assessment of the TPCs’ similarity.

## 4. Experiments

### 4.1. Experimental Environment

**Figure 11:**
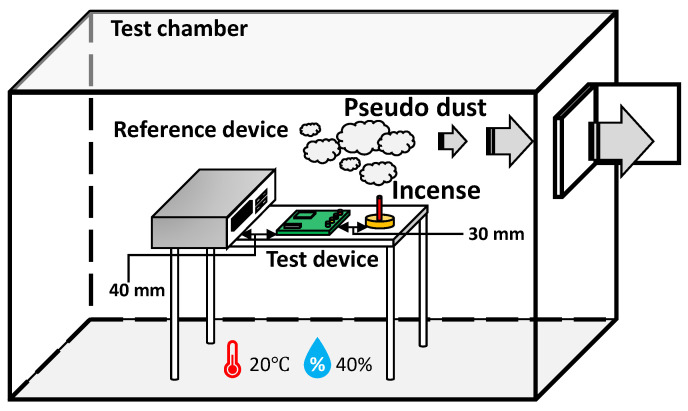
Arrangement of the two sensor devices and test environment.

In this study, two particle sensors were used as test and reference devices. Figure 11 illustrates the two devices studied in the test chamber.

The two sensors were positioned in close proximity to each other within the test chamber to enable simultaneous sensing and measurement of a stable change in particle concentration. In this study, incense smoke was utilized as a surrogate for particulate matter (PM). Following the smoking of incense for a specified duration, the process of reducing the dust concentration through ventilation was observed and measured. To assess the feasibility of digitally twinning the reference device, the temperature and humidity levels inside the chamber were maintained at a constant level to minimize the influence of external environmental variables. The test device employed in the experiment captured the ADC readings at a sampling frequency of 50 kHz, followed by the acquisition of PCs at a sampling frequency of 1/6 Hz, matching the sampling frequency of the RPCs.

### 4.2. Test Dataset Generation

**Figure 12:**
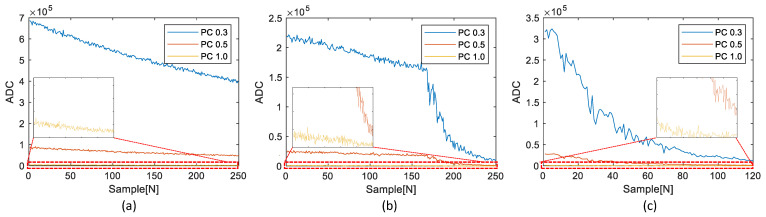
Three RPC dataset from the reference device: (**a**) first dataset with high concentration, (**b**) second dataset with ventilation rate change, (**c**) third dataset with low concentration.

For the experiments, three datasets were acquired from the chamber. The first dataset assumed a high-concentration condition; the second dataset assumed a complex dataset, including changes in the ventilation rate; and the third dataset assumed a low-concentration condition. Figure 12 shows the RPCs of the three datasets obtained from the reference device.

The small plot boxes inside each figure show the enlarged results of each PC1.0. The reference device provides seven RPCs: RPC0.3, RPC0.5, RPC1.0, RPC2.5, RPC4.0, RPC7.0, and RPC10.0. However, Figure 12 displays only the three RPCs, RPC0.3, RPC0.5, and RPC1.0, because the test device can reliably replicate these RPCs. The possibility of the reliable replication of RPC2.5 or higher RPCs was analyzed through the following experiment.

**Figure 13:**
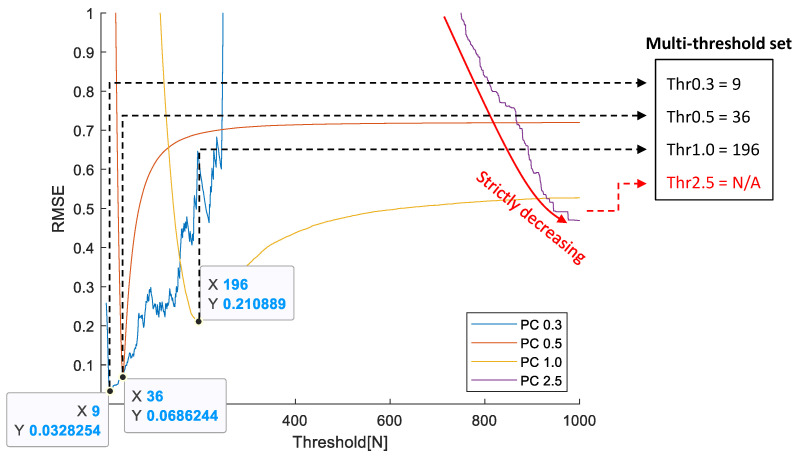
RMSE distribution of each PC according to the thresholds.

Figure 13 shows the example of the change in RMSE between the RPC and TPC obtained according to the threshold for the first dataset in Figure 12a.

In the case of TPC0.3, TPC0.5, and TPC1.0, it is possible to detect the optimal threshold where the RMSE has a minimum value. However, in the case of TPC2.5, the RMSE continuously decreases as the threshold increases, and the optimal threshold of TPC2.5 continuously increases until all TPC2.5 values become 0. This phenomenon occurs because the expected result value is less than 1 when normalizing TPC2.5 according to the ratio of RPC0.3 to RPC2.5. Each value of TPC2.5 represents the number of samples larger than the given threshold Thr2.5. Therefore, having a value less than 1 means that there are no samples larger than Thr2.5, and the RMSE is minimized when all TPC2.5 values become 0.

This limitation arises from the fact that the test device used in this study is a low-cost, single-sensing device. As a result, we observed that reliable replication of the RPC calculation algorithm is feasible for PC0.3, PC0.5, and PC1.0 using the test device. Since the focus of this study is to generate TPCs that replicate RPCs, PCs with values exceeding PC2.5 were excluded from the analysis.

### 4.3. Experiments

**Figure 14:**
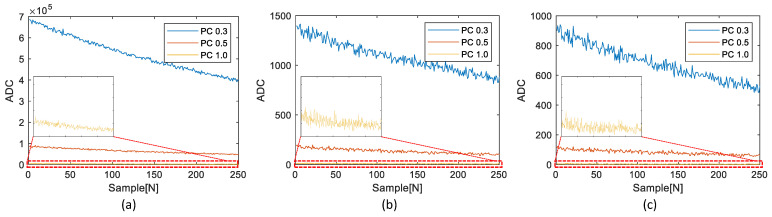
Comparison of results of the first dataset: (**a**) RPCs, (**b**) TPCs determined by the existing general multi-threshold detection, (**c**) TPCs determined by the proposed simplified multi-threshold detection.

**Figure 15:**
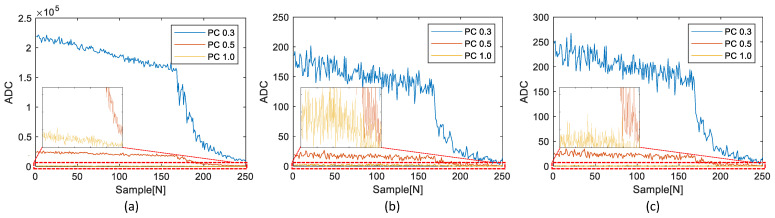
Comparison of results of the second dataset: (**a**) RPCs, (**b**) TPCs determined by the existing general multi-threshold detection, (**c**) TPCs determined by the proposed simplified multi-threshold detection.

**Figure 16:**
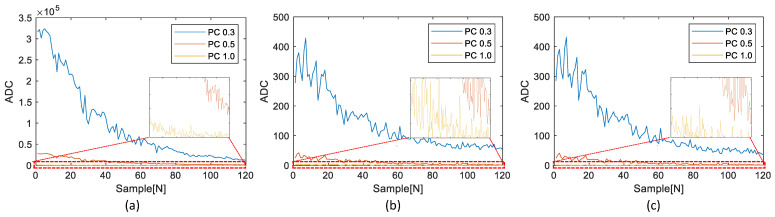
Comparison of results of the third dataset: (**a**) RPCs, (**b**) TPCs determined by the existing general multi-threshold detection, (**c**) TPCs determined by the proposed simplified multi-threshold detection.

The experimental results involved a comparison between the RPCs and the corresponding TPCs. The TPCs obtained from the test device were generated using both the existing general multi-threshold approach and the proposed simplified multi-threshold approach. Figure 14, Figure 15 and Figure 16 illustrate the comparisons of the results for the three datasets shown in Figure 12.

The small plot boxes inside each figure show the enlarged results of each PC1.0. As shown in Figure 14, Figure 15 and Figure 16, without knowledge of the ADC and PCs generation algorithm of the reference device, we replicated the RPCs and the TPCs.

Table 2 shows a detailed comparison of the RMSE results in Figure 14, Figure 15 and Figure 16.

Through our evaluation, we observed a notable improvement in the digital twinning performance, as the overall RMSE in the proposed method was significantly reduced. Specifically, the overall RMSE decreased from 0.297692 to 0.123572, indicating a substantial reduction of approximately 58.5%. Of particular significance is the improvement observed in PC1.0, where the average RMSE decreased from 0.752072 to 0.244419, representing a significant reduction in RMSE of about 67.5%. This reduction in RMSE highlights the effectiveness of the proposed method in accurately replicating PC1.0 values. These findings demonstrate the enhanced performance and accuracy achieved by our proposed method in digital twinning, providing more reliable replication of PC values and reducing the discrepancy between the reference device and the embedded device.

**Figure 17:**
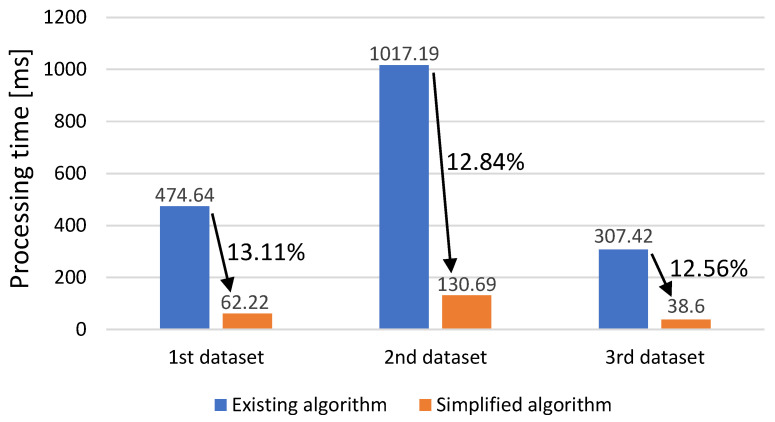
Comparison of processing time of optimal multi-threshold searching algorithms.

Figure 17 shows a comparison of the processing times of the optimal multi-threshold searching algorithms for the three test datasets.

The execution time of the simplified algorithm was reduced by an average of approximately 87% from 474.64 to 62.22 for the first dataset, as shown in Figure 14; from 1017.19 to 130.69 for the second dataset, as shown in Figure 15; and from 307.42 to 38.60 for the third dataset, as shown in Figure 16.

Compared to the existing algorithm that has to repeat the calculation of the PC for the 50 kHz sampling frequency data, the proposed simplified algorithm that requires only one calculation through the LUT can considerably reduce the execution time. Therefore, it can be confirmed that the proposed simplified algorithm using LUT not only significantly reduces the execution time, but also maintains the distribution of PCs, as shown in Figure 14, Figure 15 and Figure 16.

## 5. Conclusions

In this study, we aimed to replicate the PCs of a high-cost reference device using a low-power and low-cost embedded device with a single sensor. To optimize the execution time, we proposed a simplified multi-threshold search process for calculating TPCs using an LUT. The experimental results demonstrated that the TPCs calculated using the LUT effectively preserved the distribution of RPCs and existing TPCs while significantly reducing the execution time and RMSE by approximately 87% and 58.5% on average, respectively.

The RMSE analysis based on the LUT thresholds revealed that the proposed method reliably replicated PC0.3, PC0.5, and PC1.0 with optimal thresholds. However, in the case of PC2.5 or higher PCs, digital twinning was not feasible due to the limited number of samples in the single-sensor device compared to the reference device. To expand the applicability of digital twinning, future research should focus on increasing the number of channels or signal amplification to enable the detection of appropriate thresholds for higher PCs.

Furthermore, the proposed method has limitations in that it was tested under constant temperature and humidity conditions to minimize the influence of external factors. To achieve more generalized digital twinning, it is necessary to explore the preprocessing of RPCs and raw ADC data or post-processing of TPCs to account for changes in temperature and humidity. Developing a robust PC calculation algorithm that can handle environmental variations is crucial, along with analyzing sensor response time, PC calculation execution time, and minimum processor performance requirements for real-time processing.

The proposed algorithm successfully obtained TPCs by replicating RPCs. As a next step, future research should focus on obtaining test particulate matter (TPM) by replicating the unknown transfer function of reference particulate matter (RPM) from RPCs. Considering the scale difference between RPCs and TPCs in the TPM calculation process will ensure that RPMs and TPMs have similar scales and distributions. Since PMs are widely used as indicators of dust concentration, this future research will represent the final stage in achieving digital twinning of dust sensing systems. 

## Figures and Tables

**Table 1:** Comparison of characteristics of dust sensing methods.

Sensing Method	Accuracy	Time	Measurement	Portability	Cost
Gravimetric	Very high	24 h	Manual	Low	High
Bete-ray	high	1 h	Automatic	Low	High
Light scattering	Low	1 s ∼1 min	Automatic	High	Low

**Table 2:** RMSE comparison between existing method and proposed method.

Method	Data	RMSE			
		PC0.3	PC0.5	PC1.0	Average
Existing method	First dataset	0.046512	0.072553	0.312398	0.143821
	Second dataset	0.048505	0.095210	0.960891	0.368202
	Third dataset	0.068228	0.092008	0.982927	0.381054
	Average	0.054415	0.086590	0.752072	0.297692
Proposed method	First dataset	0.032825	0.068624	0.210889	0.104113
	Second dataset	0.046248	0.088694	0.241081	0.125341
	Third dataset	0.062146	0.080355	0.281287	0.141263
	Average	0.047073	0.079224	0.244419	0.123572

## Data Availability

Not applicable.

## Abbreviations

The following abbreviations are used in this manuscript:

ADC	Analog-to-digital converter
RMSE	Root mean square error
LUT	Look-up table
FIR filter	Finite impulse response filter
IIR filter	Infinite impulse response filter
PC	Particle count
RPC	Reference particle count
TPC	Test particle count
PM	Particulate matter
RPM	Reference particulate matter
TPM	Test particulate matter
